# Cesarean section and breastfeeding outcomes in an Indigenous Qom community with high breastfeeding support

**DOI:** 10.1093/emph/eoab045

**Published:** 2022-01-04

**Authors:** Melanie Martin, Monica Keith, Sofía Olmedo, Deja Edwards, Alicia Barrientes, Anwesha Pan, Claudia Valeggia

**Affiliations:** 1 Department of Anthropology, University of Washington, 314 Denny Hall, Box 353100, Seattle, WA 98195-3100, USA; 2 Centro de Investigaciones y Estudios sobre Cultura y Sociedad (CIECS-CONICET), Universidad Nacional de Cordoba, AV. Valparaiso S/N. Ciudad Universitaria, Cordoba, Argentina; 3 Department of Anthropology, Yale University, P.O. Box 208277, New Haven, CT 06520-8277, USA

**Keywords:** birth mode, breastfeeding duration, kin support, cesarean section, postpartum period

## Abstract

**Background and objectives:**

Cesarean section may lead to suboptimal breastfeeding outcomes, though evidence has been mixed. Factors, such as premature birth, birth weight and maternal age may independently increase risk of cesarean and hinder breastfeeding initiation, while maternal preferences, support and sociostructural barriers may influence breastfeeding practices beyond the immediate postpartum period.

**Methodology:**

We assessed impacts of cesarean section and gestational factors on breastfeeding duration among Indigenous Qom mothers in Argentina who have strong traditional breastfeeding support. We modeled transitions from exclusive breastfeeding to complementary feeding and from complementary feeding to full weaning in a Bayesian time-to-event framework with birth mode and gestational covariates (*n* = 89 infants).

**Results:**

Estimated median time to full weaning was 30 months. Cesarean-delivered babies were weaned an average of 5 months later adjusting for gestational age, maternal parity and infant sex. No factors were associated with time-to-complementary feeding, and time-to-complementary feeding was not associated with time-to-full weaning.

**Conclusions and implications:**

Among Indigenous Qom mothers in Argentina, cesarean section was not associated with suboptimal breastfeeding outcomes. Although some Qom mothers do experience early breastfeeding problems, particularly following first birth, problems are not more frequent following cesarean delivery. Traditional postpartum kin and community support during prolonged postpartum periods may be instrumental in helping mothers to overcome early breastfeeding problems due to cesarean or other risk factors.

## Lay Summary

We examined associations between cesarean section and breastfeeding transitions among Indigenous mothers in Argentina. Mothers delivering via cesarean section did not introduce complementary foods earlier, and conversely weaned later than mothers who delivered vaginally. Postpartum kin support typical of traditional birthing practices may help mothers overcome early breastfeeding problems.

## BACKGROUND AND OBJECTIVES

Cesarean sections are life-saving in many cases, but involve an evolutionary novel sequela of physiological and behavioral events that may increase long-term health risks for mothers and offspring, and are of increasing concern given rising rates of medically unnecessary cesareans [1, 2]. Cesarean section has been associated with delayed onset of lactogenesis [3, 4] and suboptimal breastfeeding outcomes, which include delayed or lower rates of breastfeeding initiation [5, 6], and ceasing exclusive or total breastfeeding durations before recommended durations of 6 and 24 months, respectively [7, 8]. However, evidence for associations between cesarean section and suboptimal breastfeeding outcomes have been mixed [9–11], which may reflect confounding between the evolutionarily novel pathways by which cesarean section influences feeding outcomes at different stages of lactation, as well as with other maternal and infant factors that influence cesarean risk and breastfeeding practices.

Directly, scheduled or unscheduled cesareans may hinder successful breastfeeding initiation due to the lack of exposure to parturient hormonal surges, infant drug exposures, maternal-infant separation, more limited skin-to-skin contact and postoperative pain and mobility limitations, which may impair latching, maternal-infant bonding, infant alertness and olfactory learning [1, 12, 13]. Early difficulties establishing breastfeeding may in turn lead mothers to cease breastfeeding altogether, or lead to early and increased reliance on complementary feeding—including formula supplementation—which may displace breast milk intake, leading to downregulated milk production and ultimately earlier than intended full weaning [14]. Indirectly, cesarean section may alter infant gut microbial colonization [15–17] and promote more rapid postnatal weight gain [18–20], which may be perceived as warranting earlier or increased complementary feeding [21, 22].

Conversely, proposed effects of cesarean sections on breastfeeding initiation and continuation may be confounded when occurring in conjunction with preterm births [23], or prolonged labor and delivery, which heightens maternal and fetal stress and impairs lactogenesis [24–26]. Factors, such as primiparity, obesity, adolescent and advanced maternal age have also been independently associated with greater risk of cesarean section [3, 27, 28] and suboptimal breastfeeding outcomes [29–31]. Mothers who opt for elective cesareans may also be less likely to intend to breastfeed [7]. Lastly, cesarean risk factors and breastfeeding outcomes may be compounded among some historically disadvantaged racial and economic groups who experience both greater sociostructural barriers to breastfeeding [8, 32] and chronically higher rates of chronic disease, psychosocial stress and maternal and fetal health risks [33, 34].

Targeted interventions may improve breastfeeding outcomes following cesarean, yet their implementation and efficacy have not been rigorously tested to date [12]. Furthermore, much research on infant outcomes following cesarean has been done in high-income populations [35], with generally shorter durations of exclusive and total breastfeeding that are influenced in part by lack of support for and barriers to breastfeeding maintenance [36]. An evolutionary-informed framework for examining associations between cesarean and breastfeeding outcomes may improve the efficacy of potential interventions by considering both the socioecological context of breastfeeding within specific populations and the proposed mechanistic effects of cesarean at specific feeding transitions—e.g. direct effects on early supplementation necessitated by early latch and supply problems vs indirect effects on earlier weaning mediated by rapid growth and increased complementary feeding that reduces breastfeeding intensity. In particular, we propose that research is needed with ethnically and culturally homogenous communities that exhibit traditionally strong kin and community support for prolonged and intensive breastfeeding. Such socioecological contexts are more representative of those that shaped breastfeeding practices during human evolution, and in effect remove confounding due to within-population variation in maternal feeding preferences and sociostructural barriers to breastfeeding. Research with Indigenous populations undergoing rapid socioeconomic change is additionally needed, as modern biomedical practices and globalization increasingly erode traditional birth and breastfeeding practices [36]. To these ends, we examined associations between cesarean sections and exclusive and total breastfeeding durations in Namqom, an Indigenous Qom community in Formosa, Argentina.

Breastfeeding is nearly universal in Namqom, with full weaning at 2 years on average [37, 38]. However, in a recent analysis of birth records collected from 90 Namqom infants [39], we observed cesarean rates of 46%, with cesarean risk increased for pre- to early term births, adolescent mothers, and mothers over 30. In a preliminary analysis in that study, cesarean section was not significantly associated with risk of full weaning after adjusting for infant age across all subjects 0–4 years. Relative risk ratios for full weaning were higher for cesarean-delivered infants before 12 months of age, however, though <10% of infants were weaned before 12 months of age [37].

In this study, we follow-up on those preliminary results with a more robust analysis to better address the potential complexity of relationships between birth mode and different feeding transitions discussed above. This exploratory analysis re-examines data from Qom mother–infant birth records and monthly feeding interviews to test if cesarean section separately associates with time-to-complementary feeding or full weaning, using a Bayesian framework that adjusts for key gestational confounds. In this analysis, we observed, on average, no difference in exclusive breastfeeding durations, and later total breastfeeding durations in cesarean- as compared to vaginally born infants, and wanted to further examine the role of postpartum kin support in overcoming any potential early problems with breastfeeding. To provide additional context for aspects of birth and breastfeeding experiences not documented in prior data collection, we conducted retrospective interviews on these topics in 2019 with a subsample of mothers from the original study. We present qualitative results from these interviews following analysis of time-to-complementary feeding and weaning. Results demonstrate the need for future causal design studies sufficient to unpack the complex pathways linking birth mode and infant feeding outcomes.

## METHODOLOGY

### Study population

The Qom (Toba) are an Indigenous population of the Gran Chaco region of South America. Formerly semi-nomadic hunter-gatherers, the Qom have experienced extensive environmental and cultural change since the mid-20th century, as government policy and habitat degradation have forced most Qom into small settlements around northeastern Argentina. Most Qom families are reliant on government welfare, with autonomy and economic opportunity limited by resource allocation and ethnic discrimination [40]. This study was conducted in Namqom (pop. ∼5000), a peri-urban community located 11 km outside of the capital city of Formosa (pop. 234 000).

The total fertility rate of Qom mothers has been previously estimated at 6.8 births, with first births commonly during adolescence [40]. Namqom mothers have access to free monthly prenatal and child wellness visits through a centrally located clinic in the community. Since the 1990s, nearly all Namqom mothers have given birth at public hospital in the city of Formosa. In this hospital, multiple mother–infant pairs room together following birth. There are no nurseries, though the hospital is equipped with a neonatal intensive care unit.

In studies carried out over the last two decades, Namqom mothers have been observed to breastfeeding on demand, introduce complementary foods and liquids (primarily powdered cow’s milk) at 4–6 months of age, and wean after 2 years [37, 38]. Namqom mothers are rarely employed outside the home; infants are observed in the care of their mothers ∼75% of the time, with fathers and female kin providing remaining care [41]. Infants of some adolescent mothers may be fostered by their maternal grandmothers or another family member. Infants are well-nourished, with average weight-for-age and height-for-age *z*-scores falling within normal WHO reference ranges (2 to −2 SD), no cases of wasting observed, and BMI-for-age *z*-scores within or greater than normal reference ranges [42]. About 46% of the infants in this study were born via cesarean section, and key gestational and birth characteristics are presented in Table 1.

**Table 1.** Sample statistics for Qom mother–infant pairs

Sample group	Original data collection	Retrospective interviews
Variable	*n* (%)	*n* (%)
Female	51 (57)	32 (59)
Male	38 (43)	22 (41)
C-section	41 (46)	22 (41)
Vaginal birth	48 (54)	32 (59)
Primiparous mother	37 (42)	17 (31)
Multiparous mother	52 (58)	37 (69)
Maternal age <20 years	37 (42)	19 (37)
Maternal age 20–29 years	36 (40)	25 (49)
Maternal age 30+ years	16 (18)	7 (14)
Low birth weight (<2.5 kg)	5 (6)	2 (4)
Normal birth weight (2.5–4.0 kg)	76 (85)	42 (84)
High birth weight (>4.0 kg)	8 (9)	6 (12)
Preterm (<37 weeks)	10 (11)	6 (12)
Early term (37–38 weeks)	26 (29)	13 (26)
Full term (39–40 weeks)	48 (54)	29 (58)
Late term (40+ weeks)	5 (6)	2 (4)

Original data collection 2011–14 (*n* = 89); retrospective interviews 2019–20 (*n* = 54).

### Prospective data collection (2011–14)

Principal data for this study were collected between 2011 and 2014 by the Chaco Area Reproductive Ecology (CARE) program, as part of a longitudinal study on infant growth and feeding transitions during weaning (NSF BCS-0952264). CARE researchers attempted to contact all Namqom families with infants <12 months of age to participate. Between 2011 and 2012, a total of 101 families meeting eligibility criteria were enrolled. Researchers visited participants in their homes monthly to collect infant anthropometric measures and breastfeeding status, defined here as exclusively breastfeeding, complementary feeding (breastfeeding with additional liquids and/or solids) or fully weaned (no longer breastfeeding). Mothers were asked about the types of foods and liquids typically given to infants, but not frequency or amounts given. Formula-feeding was not specifically assessed, though most Qom infants are bottle-fed with reconstituted powdered cow’s milk, when available. Infant formula is not readily accessible or economically feasible for most families. Birth mode, weight, length and gestational age were documented from birth records archived at the local health clinic and shared with consent from participant mothers and clinic administrators. The following variables were not available from birth records or systematically collected for inclusion in analysis: cesarean type and reason for cesarean, time to breastfeeding initiation, maternal pre-gestational weight, gestational weight gain, maternal weight at monthly interviews and subsequent pregnancy. Infant weight and weight gain were not analyzed in association with feeding transitions because many infants were already complementary feeding at enrollment. Infants were followed for a minimum of 3 months post-weaning or until study’s end in 2014. This study is restricted to 89 infants (51 females and 38 males) with complete maternal and birth data available (Table 1).

### Retrospective interviews (2019–20)

Between August 2019 and January 2020, we contacted 49 mothers who provided additional information on 54/101 (53%) of births in the original dataset. Of the remaining mothers not interviewed: 20 had moved out of the community or ceded custody of the participant child to another family member; 13 could not be located; 9 were indisposed due to death, illness, substance abuse or other conflicts; and 6 declined interviews. Mothers were asked the following about their participant children’s births, as applicable: the reason for their cesarean, if it was scheduled or unscheduled and total number of prior cesareans; if any other problems not documented in birth records were diagnosed during pregnancy; if they had any problems breastfeeding in the hospital or once at home. They were also asked questions about breastfeeding difficulties, pain and help received following their first births.

### Ethical standards

Interviews were conducted in Spanish in mothers’ own homes by trained Argentinian and Qom research assistants. Mothers provided verbal informed consent for their infants to participate. The research protocols were approved by the internal review boards of both the University of Pennsylvania (Protocol #811200) and Yale University (Protocol #1406014104 for data collected through 2014 and #2000026021 for interviews in 2019–20).

### Statistical methods

Two breastfeeding transitions were modeled using a Bayesian time-to-event analysis framework (i) exclusive breastfeeding to complementary feeding and (ii) complementary feeding to full weaning. We used the rstanarm package in R v. 4.0.3 to fit time-to-event models with full Bayesian inference [43–45]. All models ran for 4000 iterations with 4 chains each, using weakly informative priors that centered each model’s predictors internally [43]. Time-to-complementary feeding models were left- and interval-censored, and included repeated observational intervals, with a random-level effect for infant ID. Time-to-weaning models had defined bounds modeled on each infant’s last observation interval (52 weaning event intervals, 37 right-censored intervals). See online supplementary material for additional description of methods used in constructing baseline time-to-complementary feeding and time-to-weaning models.

We screened gestational covariates individually in time-to- complementary feeding and time-to-weaning models. All models included infant sex as a baseline covariate, due to previously observed higher weight-for-age in cesarean-born males in this sample [46], which may influence sex-biased feeding trajectories. A model selection screening process was then used to select between parity, maternal age, birth weight and gestational age due to potential for covariance between these terms and with birth mode [39]. Maternal age was modeled categorically due to the previously observed higher prevalence of cesarean among participant mothers <20 and >30 years old [39]. Similarly, because cesarean risk and complementary feeding initiation and intensity may have non-linear associations with birth size and gestational age, these factors were modeled three different ways during screening. Birth weight was modeled as: continuous; low (<2500 g)/normal (2500– 4500 g)/high (>4500 g); and low/normal-high. Gestational age was modeled as: continuous; pre (<37 weeks)/early (37–38 weeks)/full (39–40 weeks)/late term (41+ weeks); and pre–early/full–late term categories (Table 1).

Based upon leave-one-out cross-validation (LOO) and Watanabe–Akaike information criteria (WAIC), we selected covariates with the best model fit. Our full time-to-complementary feeding and time-to-weaning models each included sex and birth mode, along with the strongest of either parity vs maternal age, and one form of either birth weight vs gestational age. We also modeled the effect of age at complementary feeding on time-to-weaning for a down-sample of 56 infants who had closed age intervals recorded for their transitions from exclusive breastfeeding to complementary feeding. De-identified data and code are available at https://github.com/mhkeith/Qom-Breastfeeding-Transitions.

## RESULTS

### Time-to-complementary feeding

**Figure 1.**
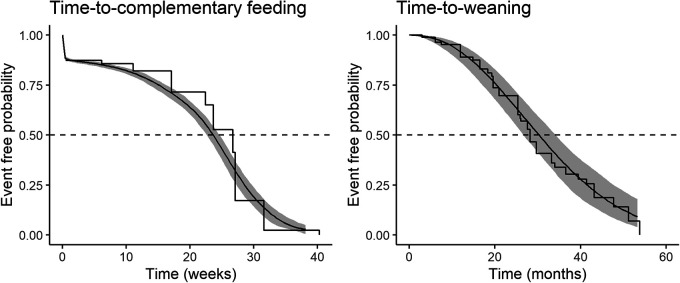
Probability curves for time-to-complementary feeding (left, Gompertz) and time-to-full weaning (right, Weibull) Smooth curves show the baseline hazard probabilities with 95% posterior uncertainty limits in gray, and the observed stepwise Kaplan–Meier curves are overlaid.

The median time-to-complementary feeding for this sample of Qom infants was estimated at 23 weeks (Fig. 1). Baseline hazard screening indicated that the Gompertz distribution best fit the time-to-CF data, and all subsequent time-to- complementary feeding models used this parametric hazard function (Supplementary Fig. S1 and Table S1). Time-to- complementary feeding models included 89 infants with 253 total observations, including repeated observations among the 57 infants who were not left-censored. The observed stepwise Kaplan–Meier curve is also plotted for comparison and shows a median age at complementary feeding of 27 weeks. We calculated the raw median ages at the start and end of the 56 closed-interval complementary feeding transitions recorded, and both modeled medians fall within this range of 21.00–27.07 weeks.

**Figure 2.**
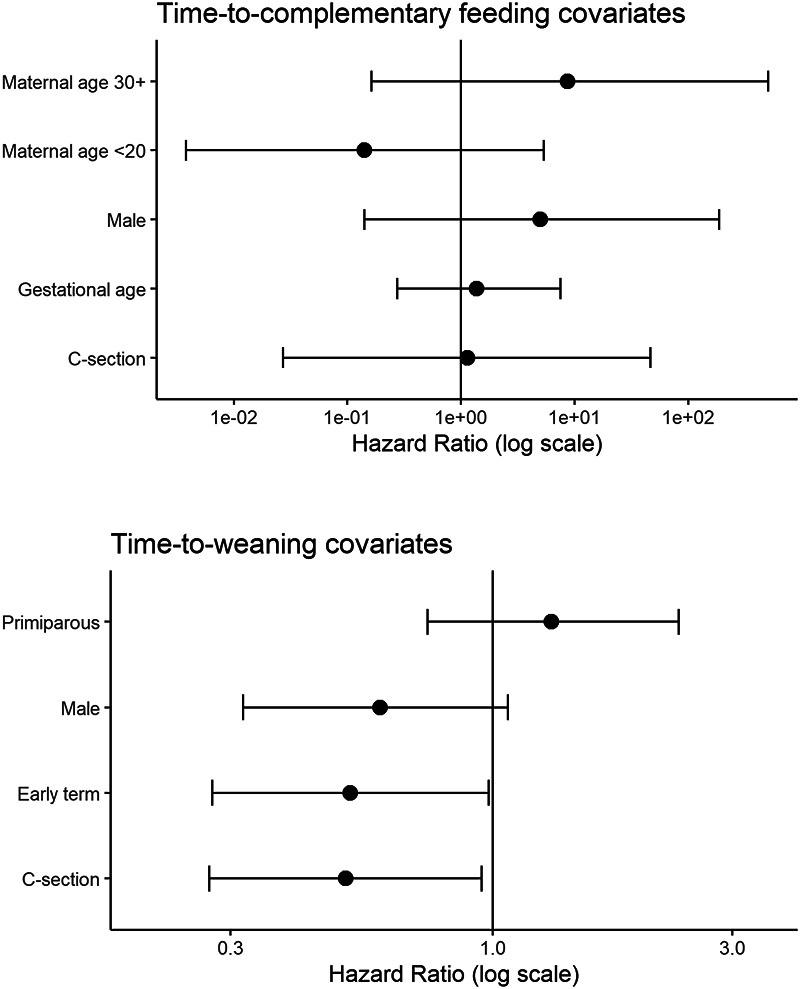
Covariate hazard ratios for complementary feeding (top) and weaning (bottom) transitions Posterior means and 95% credible intervals from full time-to-event covariate models.

None of the covariates evaluated showed a meaningful effect on the time-to-complementary feeding in individual screening models. Supplementary Table S2 reports means from each model’s posterior distribution of hazard ratios and 95% Bayesian credible intervals include 95% of the hazard ratio estimates retained from sampling the full posteriors. Effective sample sizes for these and all subsequent models are >3000, and all Gelman–Rubin statistics indicate successful chain convergence (Rhat ≈ 1.00) [47]. All hazard ratio intervals in these models span 1.0, indicating that individual parameters confer neither consistently lower nor higher hazard rates (Supplementary Table S2). Similarly, in a full model including a random-level ID effect, none of the coefficient intervals for infant sex, birth mode, maternal age and gestational age were consistently associated with earlier or later transition to complementary feeding (Table 2 and Fig. 2). Sex-adjusted Kaplan–Meier curves likewise show largely overlapping time-to-complementary feeding rates among Qom infant males and females (Supplementary Fig. S2).

**Table 2.** Posterior means and hazard ratios with 95% credible intervals for full time-to-event covariate models: time-to-complementary feeding and time-to-full weaning

		Coefficient estimate		Hazard ratio	
Model	Variable	Post.mean	95% cred.int.	Post.mean	95% cred.int.
Time-to-CF					
	Male	1.588	(−1.957, 5.159)	4.893	(0.141, 173.928)
	C-section	−0.007	(−3.561, 3.784)	0.993	(0.028, 43.991)
	Maternal age <20	−1.778	(−5.497, 1.927)	0.169	(0.004, 6.866)
	Maternal age 30+	2.141	(−2.115, 6.250)	8.508	(0.121, 518.078)
	Gestational age	0.303	(−1.375, 2.031)	1.354	(0.253, 7.624)
Time-to-full weaning					
	Male	−0.517	(−1.120, 0.068)	0.597	(0.326, 1.070)
	C-section	−0.671	(−1.293, −0.045)	0.511	(0.274, 0.956)
	Primiparous	0.274	(−0.305, 0.866)	1.316	(0.737, 2.377)
	Early term (<39 weeks)	0.645	(0.019, 1.255)	1.907	(1.019, 3.507)

### Time-to-weaning

**Figure 3.**
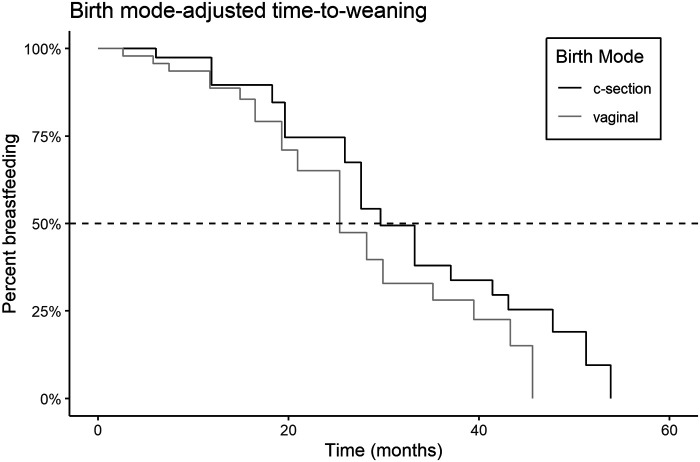
Kaplan–Meier time-to-full weaning curves adjusted by birth mode

The estimated median time-to-weaning was 30 months. The Weibull distribution best fit the time-to-weaning data according to LOO and WAIC, and was used in all subsequent time-to-weaning models (Supplementary Fig. S3 and Table S1). The parametric hazard estimate, based on each infant’s last data interval (*n* = 89, 52 weaning event intervals and 37 right-censored), was similar to the Kaplan–Meier estimated median of 28 months (Fig. 3). Based only on the 52 closed-interval weaning transitions recorded, we calculated raw median age at weaning between 24.59 and 25.73 months, noting that modeled medians are additionally informed by 37 right-censored age intervals during which some infants continued complementary feeding through later ages.

When modeled individually, none of the screened gestational covariates were consistently associated with time-to-weaning (Supplementary Table S2). Likewise, the down-sampled complementary feeding covariate model did not show a consistent association between age at complementary feeding and time-to-weaning. All hazard ratio credible intervals span 1.0, however, the time-to-weaning data produces much smaller parameter estimate intervals than the time-to- complementary feeding data since there are no left-censored weaning events.

Several covariates were consistently associated with time-to-weaning in a full model that included infant sex, birth mode, parity and gestational age (early and full-term categories) covariates (Table 2 and Fig. 2). When controlling for both maternal and gestational age group, cesarean-born infants have lower time-to-weaning event rates, indicating later weaning. Kaplan–Meier curves show a median age at weaning 5 months later for cesarean-born infants (Fig. 3). As compared to full-term infants, pre- to early term infants have increased hazard ratios (Fig. 2 and Table 2), with a median time-to-weaning ∼4 months sooner as shown in the adjusted Kaplan–Meier plot (Supplementary Fig. S4). Although the hazard ratio interval for male infants does span 1.0, males show mostly lowered weaning event hazards (Fig. 2 and Supplementary Table S2). Sex-adjusted Kaplan–Meier curves show median time-to-weaning ∼3 months later for males than females, though curves intersect at several points before 20 months of age (Supplementary Fig. S5).

### Context of birth and breastfeeding from retrospective interviews

Cesarean rates were similar among the 54 mother–infant pairs interviewed retrospectively in 2019 (41%) and in the original sample (46%). Among cesarean-born infants in the retrospective sample, 7/22 (32%) were unscheduled and 15/22 (68%) scheduled surgeries. Only two mothers could not recall a specific reason for their cesareans. Reasons for unscheduled cesarean reported by six mothers (with some giving multiple reasons) were: protracted labor (3); premature birth (3); nuchal cord (1); placenta previa (1); high blood pressure (1). Specific reasons reported by 14 mothers for scheduled cesarean were: high blood pressure (6); previous cesarean (5); breech presentation (4); low blood pressure (2); adolescent maternal age (1); gestational diabetes (1); and anemia (1). Nine mothers in total had at least one previous cesarean.

Current hospital policy is supportive of breastfeeding and infants are not routinely provided formula unless medically indicated, however, we do not know what standard practices were original data collection in 2011–14. We asked participants if they or anyone else supplemented their infants with formula while in the hospital. Eight mothers specifically reported difficulties breastfeeding while in the hospital, though only four supplemented with formula. Ten mothers in total reported in-hospital formula-feeding: two delivered via unscheduled cesareans, two via scheduled cesarean and six vaginally. Seven were primiparous. Reasons reported for supplementation were: little or no milk (3); baby did not want to nurse (2); premature birth (2); nipples ‘unprepared’ for nursing (1); high bilirubin levels (1); and maternal antibiotics (1). All delivered via unscheduled cesarean; two were primiparous and two multiparous. One reported her milk did not come in for 3 days because the baby was premature, one reported general difficulty, and one reported difficulty because she was alone. The last of these mothers reported a lot of neck pain because of her high blood pressure, which prevented her from turning her neck. She also reported that her milk did not come in for 2 days, during which time she did not give the baby a bottle or formula, but was advised by a nurse to ‘drink a lot of water, milk, and soup’ so that her milk would come in. Only one mother who reported problems breastfeeding in the hospital (specifically that her nipples were ‘not formed’ and her baby could not latch) continued to have problems once at home. She reported giving him powdered milk for another week and ‘then he nursed’. No other mothers reported any problems breastfeeding once at home, but we did not ask follow-up questions about the duration of problems experienced or steps taken to resolve them. For infants whose mothers reported breastfeeding problems while in the hospital, mean ages of complementary feeding and weaning (from first month observed in 2011–14 interviews) were 21.0 ± 9.1 weeks (*n* = 8) and 30 ± 20 months (*n* = 5), respectively. For infants given formula in the hospital, mean ages were 19.8 ± 9.6 weeks (*n* = 8) and 30.1 ± 16.4 months (*n* = 6). These ages are similar to median ages estimated in the full sample (23 weeks and 30 months), and importantly suggest that early problems or supplementation did not continue, or continue frequently enough, to be reported as complementary feeding during monthly interviews or lead to substantially earlier full weaning.

A total of 48 mothers responded to questions about breastfeeding experiences following their first births (6 were study participant children). Most mothers (34/48, 71%) reported receiving help breastfeeding for the first time from one or more of the following people: their own mothers (17/34); a female relation or in-law (14/34); and hospital nurses or doctors (8/34). One participant reported that her mother, aunt and mother-in-law were with her in the hospital the whole time after the birth and helped her to nurse and position the baby. She further remarked that they spent more time with her than usual because of her cesarean, but had since heard that family members no longer can stay with mothers in the hospital. A mother who reported not having any help nevertheless remarked that she ‘learned to nurse quickly’ because she had seen her ‘mom, neighbors, and other women doing it’. Most mothers (25/48, 52%) found it difficult to breastfeed their first child, and 90% (41/48) reported that it hurt. Reasons given for difficulties were: not knowing how or anxiety about breastfeeding (12); pain (5); baby not wanting to breastfeed (3); nipples were ‘unformed’ or ‘unprepared’ (2); and not having any milk (2). Mothers described extreme discomfort breastfeeding their first children, including: cracked, chapped, painful and bleeding nipples, ‘burning’, ‘everything hurt’ and ‘like your skin being peeled off’. One mother remarked: ‘Es peor que las contracciones, se te rajan los pezones’ (‘It’s worse than the contractions, your nipples will split apart’). Another described ‘cutting’ pain that hurt for a month, but got better as she got used to it, ‘nursing him every day’.

## CONCLUSIONS AND IMPLICATIONS

In this sample of Indigenous Qom mothers from a community with strong breastfeeding support, we did not find evidence of an association between cesarean and suboptimal breastfeeding outcomes. Cesarean was not associated with time-to-complementary feeding introduction but was conversely associated with later full weaning. While a minority of mothers retrospectively interviewed (14/54) reported having problems breastfeeding or needing to supplement with formula while in the hospital, these problems were reported with near equal frequency for cesarean and vaginally delivered infants, and only one of those mothers reported continued problems at home. Furthermore, nearly all infants breastfed for at least 1 year, with more than half weaned after 2 years. Although the high cesarean rate in this community is concerning, and we cannot rule out associations between cesarean and breastfeeding problems due to limitations of our study, our findings support the contention that physical, psychological and emotional support for breastfeeding outside of the hospital is critical to long-term breastfeeding success—whether following cesarean [12, 48] or in relation to other maternal and infant risk factors [49].

Previous studies have shown that associations between cesarean and longer-term breastfeeding outcomes are driven by early breastfeeding practices. Equivalent breastfeeding outcomes have been observed for cesarean and vaginally born infants when breastfeeding was initiated and maintained for 5 days in a study of urban Chinese mothers [13], and for at least 1 month in a national sample of Mexican mothers [50]. Among Maya mothers in Yucatan, Mexico, cesarean has been associated with delayed breastfeeding initiation, but total breastfeeding durations for infants delivered via cesarean are similar or longer than those of vaginally delivered infants [36, 51]. Psychosocial support during the immediate postpartum period has been associated with higher rates of exclusive breastfeeding at 1 month [52], and may play a role in breastfeeding outcomes in this community. Qom mothers in our study were not immune to breastfeeding problems, particularly following first births, but received help and direct instruction in learning to breastfeeding from their mothers and other women kin. Similarly, first-time Himba mothers in Namibia commonly report problems latching, low milk supply, pain and anxiety about breastfeeding, but receive direct assistance from their mothers and other women [53]. Their mothers will often co-reside with them for an extended period, constantly assisting with positioning and latching, and telling them when to nurse—which reportedly enabled new mothers to overcome breastfeeding difficulties within a few days. These narratives underscore the fact that emotional and instrumental support from kin and other community members are likely integral to the intensive breastfeeding practices characteristic of the human species, similarly as human labor and birth likely evolved in conjunction with availability of emotional support and assistance [1], and co-operative breeding has been integral to the evolution of human demography and life history traits [54].

The fact that neither cesarean nor any other measured variables were associated with time-to-complementary feeding may suggest limitations of this outcome variable as a proxy for breastfeeding problems or changes in breastfeeding intensity. First, our model of time-to-complementary feeding assumes that cesarean is directly or indirectly related to early and persistent problems breastfeeding that necessitate supplementation (including bottle-feeding), which mothers in turn would report in monthly interviews. However, these assumptions do not hold if breastfeeding and supportive postpartum care help to resolve problems and mothers to revert to exclusive breastfeeding. Second, complementary feeding as reported in monthly interviews may conflate transitional or intermediate feeding (e.g. providing small tastes of food or liquids) with more energetically substantial bottle and solid food feeding that supplants milk intake. High rates of breastfeeding to 1 year or more are observed in many low and middle-income countries that have correspondingly low rates of exclusive breastfeeding to 6 months [55], suggesting that early complementary feeding may not lead to substantially reduced breastfeeding intensity in populations in which prolonged breastfeeding is normative. The lack of association between age at complementary feeding and full weaning in this study casts further doubt on the link between these two events in gradual weaning populations. Determining associations between cesarean and long-term breastfeeding outcomes would ultimately require longitudinal assessment of differences or changes in breastfeeding intensity (e.g. suckling magnitude, frequency and effort), which are notoriously difficult to operationalize across populations, particularly those that do not practice scheduled nursing [56].

The later time to full weaning observed in association with cesarean was unexpected, given that cesarean may be associated with more rapid growth and therefore earlier weaning. However, infant growth and feeding transitions are reciprocal processes that vary across contexts [57]. It is possible that Qom mothers respond to rapid growth or greater perceived needs of cesarean-born infants by breastfeeding for longer durations, as was suggested following similar observations in Yucatan Mayan mothers [51]. We further caution that the 5-month difference in estimated full weaning ages between cesarean and vaginally born infants is driven largely by breastfeeding after 2 years of age (Fig. 3). Although breast milk can be an important source of nutritional or immunological buffering throughout lactational stages, little research has been conducted on the nutritional or immunological benefits at later stages. Later breastfeeding in Qom males may reflect nurturing, non-nutritive suckling, as has been observed in chimpanzees [58, 59] and other human populations that practice gradual weaning [60]. Sex differences in weaning ages have been observed in other human populations, but are varyingly male and female-biased due to context-specific factors affecting maternal condition and energetic costs of nursing male and female offspring [61]. However, we do not know if later full weaning among Qom males reflects energetically substantial sex-biased breastfeeding investment or differences in perceived emotional needs of male and females. Future research with this population should more robustly examine breastfeeding intensity at later ages, as well as differences related to birth mode, infant sex, growth trajectories and maternal perceptions of and responses to suckling demands.

We acknowledge limitations in our analytical approach that do not sufficiently account for the complex pathways linking cesarean and breastfeeding outcomes at different lactational stages. We could not fully account for reasons for cesarean or key variables influencing cesarean risk or breastfeeding outcomes, such as maternal obesity, infant growth trajectories, maternal perceptions of milk sufficiency and infant growth or subsequent pregnancy. In addition, our analysis treated gestational age, birth weight and other gestational risk factors (maternal age and primiparity) as equivalent control variables due to potential confounding with both cesarean and breastfeeding practices, whereas a detailed causal analysis may warrant different treatment of these variables. For example, gestational age or birth weight may be intermediate variables in the causal pathways between primiparity and cesarean, primiparity and breastfeeding outcomes or cesarean and breastfeeding outcomes. Adjusting for control variables that are actually intermediate variables may underestimate the total effect of the exposure on the outcome [62]. Identifying independent and direct effects of cesarean on breastfeeding outcomes may ultimately require detailed causal analysis with alternate models that consider alternate pathways by which maternal and infant risk factors are antecedents of, confounded with, or intermediate between cesarean and early breastfeeding problems. While we lacked the sample size and data to sufficiently examine such pathways in this study, our findings underscore the need for future causal studies to include qualitative breastfeeding experiences within path analyses, and from the both the immediate neonatal through the extended postpartum period.

To conclude, we note that the global rate of cesarean deliveries has more than doubled since the 1990s [63], with the fastest increase occurring in Latin America [64]. In many Latin American populations, breastfeeding practices have been negatively impacted by the medicalization of birth and other globalizing influences, including women’s labor participation, marketing of feeding substitutes and sexualization of breasts [46]. Importantly, the estimated median time to complementary feeding (23 weeks) and full weaning (30 months) observed for Qom mothers in this study suggests that average exclusive and total breastfeeding durations in Namqom have remained relatively constant over the last 20 years [37, 38] and seemingly have not been adversely affected by hospital practices or the alarmingly high cesarean rates in this community. We attribute this success to continuing normalization of intensive breastfeeding practices, lack of socioeconomic barriers to breastfeeding and the ample support that new mothers receive from their mothers and other kin during the extended postpartum period. Public health policies and clinical practices that serve the Qom and other Indigenous communities globally should formally recognize and support birth and postpartum traditions that promote existing intensive breastfeeding practices. In populations with more substantial sociostructural barriers to breastfeeding, implementing programs and policies that provide or allow for increased postpartum social and emotional support may help to improve breastfeeding outcomes more generally and following cesarean.

## Supplementary Material

eoab045_Supplementary_Data
