# Supplementary material for: Cesarean section and breastfeeding outcomes in an Indigenous Qom community with high breastfeeding support
Source: Evol Med Public Health. 2022 Jan 4;10(1):36–46. doi: 10.1093/emph/eoab045 (PMC8830290; doi:10.1093/emph/eoab045)

**Supplementary Information**

# Supplementary Text

### Construction of baseline time-to-CF and time-to-weaning models

All of our time-to-feeding transition data is interval-censored, as mothers were interviewed approximately once a month between 2011-2014 about their infant’s BF status and exact event times were not observed. An infant’s first recorded interval of CF or weaning was used to mark these transitions. Thirty-six percent of infants (32/89) were already CF at first observation, and are left-censored in the time-to-CF models. All infants were still BF at first observation, so there are no left-censored time-to-weaning events. One infant was right-censored in time-to-CF models, whereas 42% (37/89) were right-censored in time-to-weaning models (families moved away, withdrew from the study, or the study ended before the feeding transition was reported). Breastfeeding transition events were designated as interval-censored or not observed (e.g. right-censored) as described above, and the contribution of each datapoint to each model’s likelihood depended upon its type of censoring (Goodrich et al. 2020)

We fit standard parametric (exponential, Weibull, Gompertz), flexible parametric (M-spline), and accelerated failure time (Weibull-AFT) baseline hazard models for time-to-CF (time = infant age in weeks) and time-to-weaning (time = infant age in months). Parametric methods model the rate of the event (i.e. the hazard) as a function of time, whereas the event time itself is modeled as the outcome in the accelerated failure time framework (Bradburn et al., 2003). All models included infant sex as a baseline covariate. We utilized repeated observations for infants who were not left-censored in the time-to-CF models in order to mitigate uncertainty from the combination of left- and interval-censoring in the CF data. Left-censored infants had only one time-to-CF feeding observation (the age at their first observation), whereas the other 57 infants had between 2-8 datapoints each. Time-to-CF models included a random-level effect to group datapoints by infant ID, and the repeated measures add informative, time-bounded intervals to mitigate the left-tailed skew produced from the open event intervals among left-censored infants. In contrast, all time-to-weaning event intervals had defined bounds, and time-to-weaning was modeled on each infant’s last observation interval (52 weaning event intervals, 37 right-censored intervals). We compared baseline hazard distributions for the time-to-CF and time-to-weaning models described above using leave-one-out cross-validation (LOO) and Watanabe-Akaike information criteria (WAIC) (Vehtari et al., 2017) and used the baseline hazard distributions that best fit each set of time-to-event data for all subsequent models and covariate screening.

References

Goodrich, B, J Gabry, I Ali, and S Brilleman. *Rstanarm: Bayesian Applied Regression Modeling via Stan* (version R package version 2.21.1), n.d. <https://mc-stan.org/rstanarm>.

M J Bradburn, T G Clark, S B Love, & D G Altman. (2003). Survival Analysis Part II: Multivariate data analysis – an introduction to concepts and methods. British Journal of Cancer, 89(3), 431-436

Vehtari, Aki, Andrew Gelman, and Jonah Gabry. “Practical Bayesian Model Evaluation Using Leave-One-out Cross-Validation and WAIC.” *Statistics and Computing* 27, no. 5 (September 1, 2017): 1413–32. <https://doi.org/10.1007/s11222-016-9696-4>.

# Supplementary Tables

**Table S1.** Baseline hazard screening model fit statistics. Leave-one-out information criteria (LOOIC) and Watanabe-Akaike information criteria (WAIC) for complementary feeding and weaning transition models.

|  | **Time-to-CF** | | **Time-to-weaning** | |
| --- | --- | --- | --- | --- |
| **Hazard** | LOOIC | WAIC | LOOIC | WAIC |
| Exponential | 542.2 | 515.6 | 453.4 | 453.4 |
| Weibull | 303.7 | 249.4 | 416.3 | 416.3 |
| Gompertz | 187.9 | 134.7 | 420.0 | 419.9 |
| M-spline | 334.3 | 300.4 | 418.7 | 418.6 |
| Weibull-AFT | 253.9 | 203.6 | 416.4 | 416.3 |

**Table S2**. Parameter estimates and hazard ratios for full time-to-event covariate models. Posterior means and 95% credible intervals for complementary feeding (CF) and weaning transitions.

|  |  | **Coefficient estimate** | | **Hazard ratio** | |
| --- | --- | --- | --- | --- | --- |
| **Model** | **Variable** | post.mean | 95% cred. interval | post.mean | 95% cred. interval |
| Time-to-CF | Male | 1.588 | (-1.957, 5.159) | 4.893 | (0.141, 173.928) |
| Time-to-CF | C-section | -0.007 | (-3.561, 3.784) | 0.993 | (0.028, 43.991) |
| Time-to-CF | Maternal age <20 | -1.778 | (-5.497, 1.927) | 0.169 | (0.004, 6.866) |
| Time-to-CF | Maternal age 30+ | 2.141 | (-2.115, 6.250) | 8.508 | (0.121, 518.078) |
| Time-to-CF | Gestational age | 0.303 | (-1.375, 2.031) | 1.354 | (0.253, 7.624) |
| Time-to-weaning | Male | -0.517 | (-1.120, 0.068) | 0.597 | (0.326, 1.070) |
| Time-to-weaning | C-section | -0.671 | (-1.293, -0.045) | 0.511 | (0.274, 0.956) |
| Time-to-weaning | Primiparous | 0.274 | (-0.305, 0.866) | 1.316 | (0.737, 2.377) |
| Time-to-weaning | Early term (<39 wks) | 0.645 | (0.019, 1.255) | 1.907 | (1.019, 3.507) |

# Supplementary Figures

**Figure S1.** Baseline hazard estimates for time-to-complementary feeding. Posterior medians and 95% uncertainty limits for hazard screening models (exponential, parametric Weibull, Gompertz, M-spline, and Weibull-accelerated time failure).


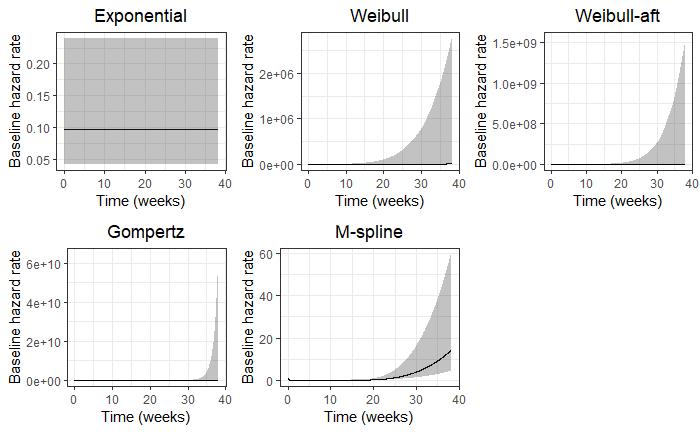


**Figure S2**. Kaplan-Meier time-to-complementary feeding curves adjusted by sex.


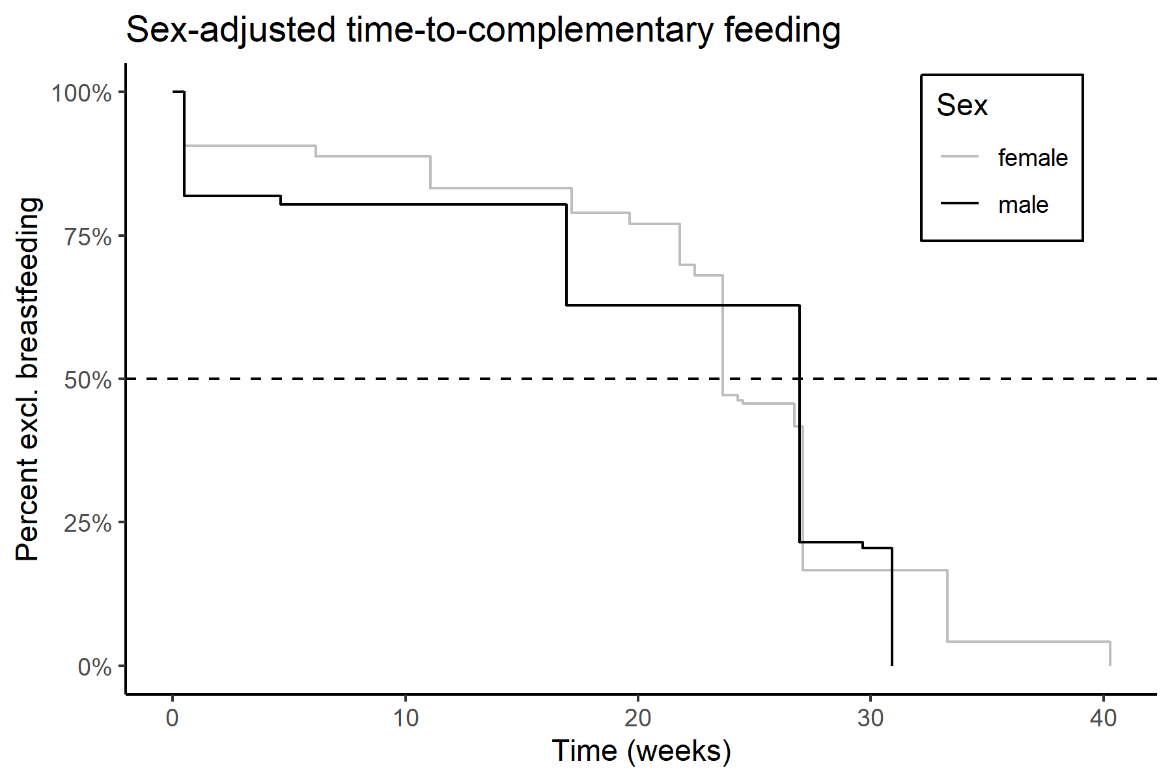


**Figure S3.** Baseline hazard estimates for time-to-weaning. Posterior medians and 95% uncertainty limits for hazard screening models (exponential, parametric Weibull, Gompertz, M-spline, and Weibull-accelerated time failure).


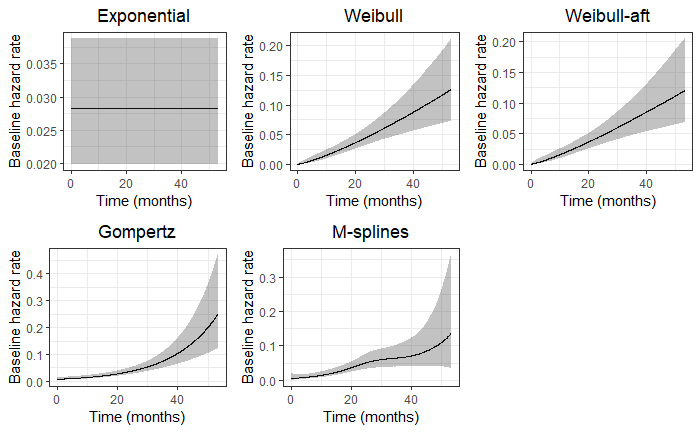


**Figure S4.** Kaplan-Meier time-to-weaning curves adjusted by gestational age (early term <39 weeks, full term ≥ 39 weeks).


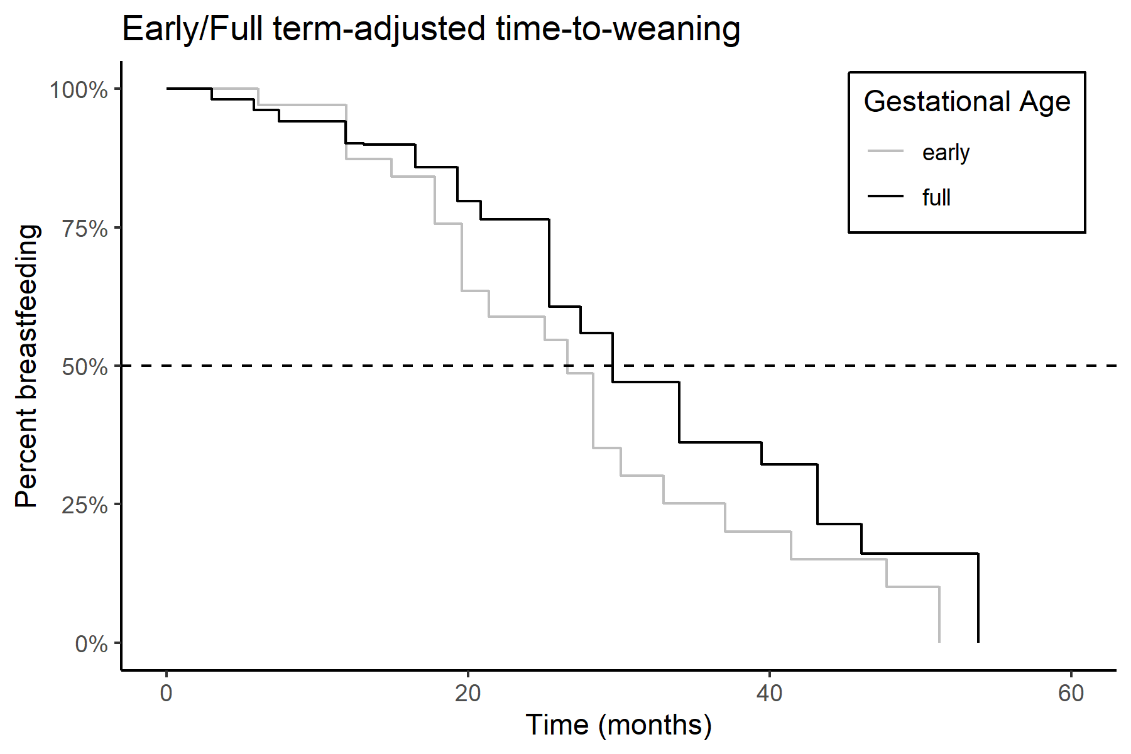


**Figure S5.** Kaplan-Meier time-to-weaning curves adjusted by sex.


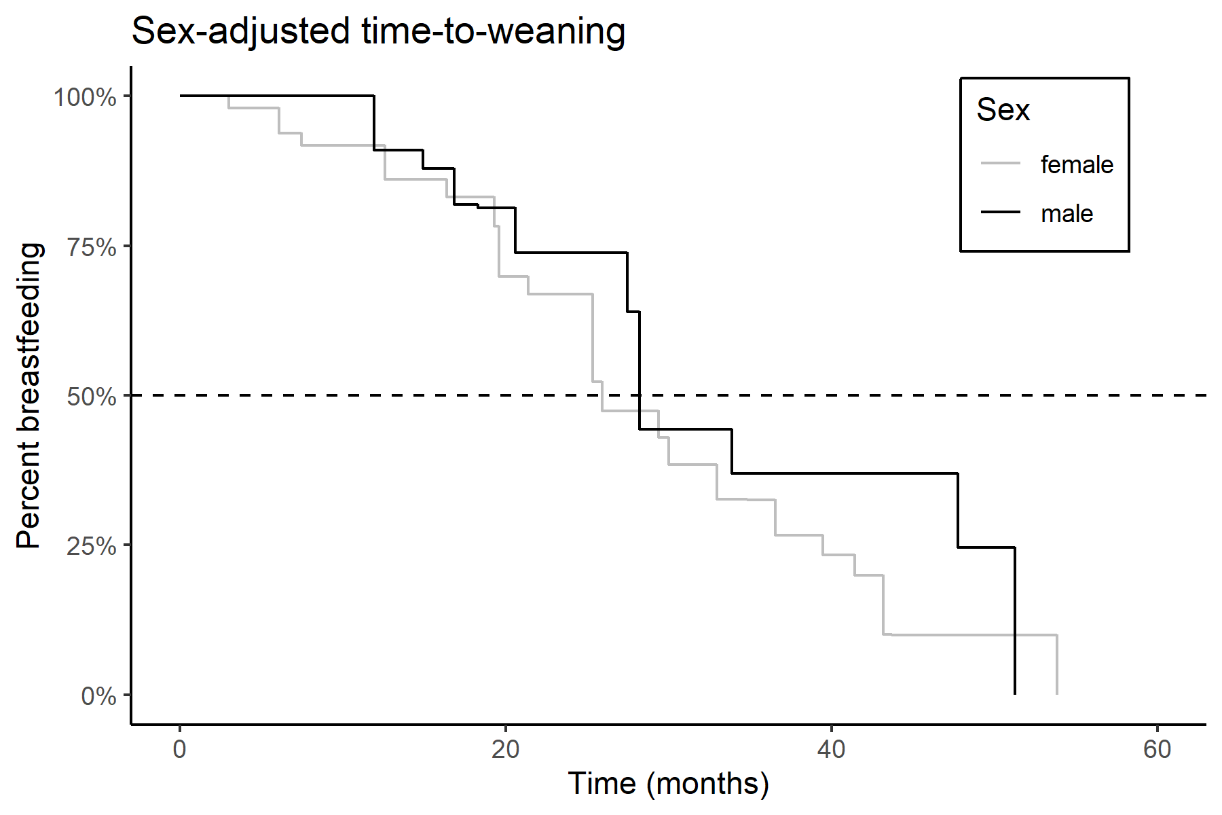

Supplement: eoab045_Supplementary_Data [file eoab045_Supplementary_Data.doc]
